# A Comprehensive Review of Intimate Partner Violence During Pregnancy and Its Adverse Effects on Maternal and Fetal Health

**DOI:** 10.7759/cureus.39262

**Published:** 2023-05-20

**Authors:** Sristy Agarwal, Roshan Prasad, Saket Mantri, Rashi Chandrakar, Shalvi Gupta, Vaishnavi Babhulkar, Samriddhi Srivastav, Arpita Jaiswal, Mayur B Wanjari

**Affiliations:** 1 Medicine, Jawaharlal Nehru Medical College, Datta Meghe Institute of Higher Education and Rersearch, Wardha, IND; 2 Medicine and Surgery, Jawaharlal Nehru Medical College, Datta Meghe Institute of Higher Education and Research, Wardha, IND; 3 Medicine, Jawaharlal Nehru Medical College, Datta Meghe Institute of Higher Education and Research, Wardha, IND; 4 Surgery, Jawaharlal Nehru Medical College, Datta Meghe Institute of Higher Education and Research, Wardha, IND; 5 Medicine, Jawaharlal Nehru Medical College, Datta Meghe Institute of Higher Education and Reserach, Wardha, IND; 6 Obstetrics and Gynaecology, Jawaharlal Nehru Medical College, Datta Meghe Institute of Higher Education and Research, Wardha, IND; 7 Research and Development, Jawaharlal Nehru Medical College, Datta Meghe Institute of Higher Education and Research, Wardha, IND

**Keywords:** prevention, adverse effects, fetal health, maternal health, pregnancy, intimate partner violence (ipv)

## Abstract

Intimate partner violence (IPV) is a significant public health issue that affects many women, including pregnant women. The aim of this comprehensive review is to examine the prevalence of IPV during pregnancy and its adverse effects on maternal and fetal health. IPV during pregnancy can take various forms, including physical, sexual, emotional, and financial abuse. The consequences of IPV during pregnancy can be severe, with adverse effects on maternal and fetal health including an increased risk of preterm birth, low birth weight (LBW), fetal injury, maternal depression, anxiety, post-traumatic stress disorder (PTSD), and even maternal death. Identifying women experiencing IPV during pregnancy and providing appropriate support and care can help mitigate the adverse effects on maternal and fetal health. The review also discusses various interventions and strategies that can be used to prevent IPV during pregnancy, such as screening and counseling for IPV, training healthcare providers to identify and manage IPV during pregnancy, and providing resources and support for women who experience IPV. Overall, the review highlights the need for increased awareness, research, and resources to prevent and address IPV during pregnancy and to promote the health and well-being of women and their infants.

## Introduction and background

Intimate partner violence (IPV) is a significant public health concern that affects millions of women worldwide. According to the World Health Organization, one in three women worldwide has experienced physical or sexual violence at the hands of an intimate partner. IPV during pregnancy is particularly concerning due to the potential adverse effects on maternal and fetal health [1,2].

IPV during pregnancy is defined as any physical, sexual, or psychological harm inflicted by a current or former partner during pregnancy or within the first year after delivery. The prevalence of IPV during pregnancy varies across different countries and cultures, with rates ranging from 2% to 35%. It is important to note that IPV during pregnancy often goes unreported and undetected, so the actual prevalence may be higher than reported [3,4].

IPV during pregnancy has been associated with a range of adverse health outcomes for both the mother and the developing fetus. Physical violence during pregnancy can result in injuries such as bruises, fractures, and head trauma. Psychological abuse during pregnancy can cause depression, anxiety, and post-traumatic stress disorder (PTSD). Additionally, pregnant women who experience IPV are more likely to experience reproductive health complications, such as sexually transmitted infections (STIs), unintended pregnancy, and induced abortion [5-7].

Adverse effects of IPV during pregnancy on fetal health can include low birth weight (LBW), preterm birth, fetal injury, and even fetal death. Children born to mothers who experience IPV during pregnancy may also have long-term health consequences, such as developmental delays, behavioral problems, and chronic health conditions [8,9].

This review article provides a comprehensive overview of the current literature on IPV during pregnancy and its adverse effects on maternal and fetal health. We will explore the types and prevalence of IPV during pregnancy, the adverse effects on maternal and fetal health, risk and protective factors, and screening and intervention strategies. By providing a detailed overview of the available research, we hope to raise awareness of the impact of IPV during pregnancy on maternal and fetal health and help healthcare providers, policymakers, and researchers develop effective prevention and intervention strategies. It is important to address IPV during pregnancy as a public health concern to ensure the health and safety of pregnant women and their babies.

## Review

Methodology

The review is based on a comprehensive search strategy that involves identifying high quality articles using relevant keywords like "Intimate partner violence (IPV)", "Pregnancy," "Maternal health," "Fetal health," "Adverse effects," "Prevention" applying filters, reviewing abstracts and full texts, and conducting a manual search of reference lists. The articles were screened for relevance and eligibility based on inclusion and exclusion criteria. The inclusion criteria required that the articles be published in the English language, report on types of IPV during pregnancy, report on the adverse effects of IPV during pregnancy on maternal health as well as fetal health, associated risk and protective factors, and potential interventions, report on both observational and interventional studies, be published from 2000 to present, and not be duplicates. Exclusion criteria included articles that were published in a language other than English, were not peer-reviewed, or were published before our selected time frame.

**Figure 1:**
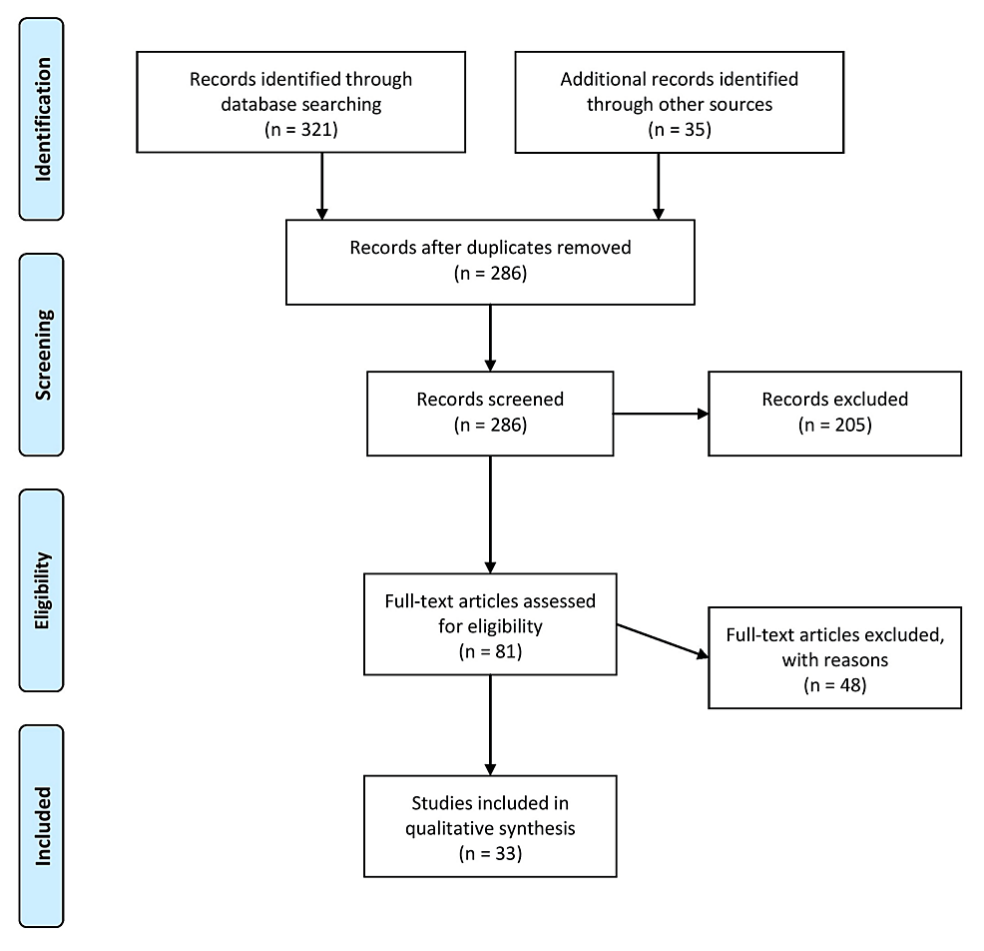
The selection process of articles used in this study. Adopted from the Preferred Reporting Items for Systematic Reviews and Meta-Analyses (PRISMA).

The article includes an introduction, sections on the types of IPV during pregnancy, adverse effects on maternal and fetal health, risk factors and protective factors for IPV during pregnancy, screening and intervention for IPV, and a conclusion with recommendations for interventions. The limitations of the review include language, as only articles in the English language were included, time restrictions, and potential biases in the selection and extraction of data. Figure 1 describes the selection process for the articles used in our study.

Types of IPV during pregnancy

Intimate partner violence during pregnancy can take many different forms, including physical violence, sexual violence, and psychological and emotional abuse.

Physical violence during pregnancy is a type of IPV that can include hitting, slapping, kicking, punching, and pushing. Pregnant women who experience physical violence are at increased risk of pregnancy complications such as vaginal bleeding, premature rupture of membranes, and preterm labor [10]. Additionally, physical violence during pregnancy can result in physical injuries to the mother and can be life-threatening in severe cases. Such violence can lead to long-term physical disabilities, chronic pain, and emotional trauma, affecting the mother's quality of life and maternal-infant bonding [11].

Sexual violence during pregnancy refers to any sexual act that is unwanted or forced by a partner during pregnancy. This type of IPV can lead to unwanted pregnancy, STIs, and other physical injuries. Sexual violence during pregnancy can also have serious psychological consequences, including anxiety, depression, and post-traumatic stress disorder (PTSD). Such trauma can negatively impact the woman's ability to bond with her baby and cope with the demands of motherhood, leading to long-term mental health issues [10,11].

Psychological and emotional abuse during pregnancy can include verbal abuse, intimidation, and isolation. It can also involve threatening behaviors, such as threatening to harm the woman or her baby. Psychological and emotional abuse during pregnancy can have a significant impact on the woman's mental health and can lead to depression, anxiety, and PTSD. This type of IPV can also negatively affect the mother-infant relationship and the development of the baby, leading to long-term social, emotional, and cognitive issues for the child [10,11].

It is crucial for healthcare providers to recognize the different forms of IPV during pregnancy, including physical, sexual, psychological, and emotional abuse. Appropriate screening and intervention can help identify women experiencing IPV and provide them with the necessary resources and support. It is also essential to raise awareness of the negative impacts of IPV during pregnancy and to develop effective prevention and intervention strategies to promote the health and well-being of pregnant women and their babies [10,11].

Adverse effects of IPV during pregnancy on maternal health

Intimate partner violence during pregnancy can have significant adverse effects on the health of pregnant women. The following are some of the most commonly reported adverse effects of IPV during pregnancy on maternal health.

Physical Injuries

Physical violence during pregnancy can result in a range of physical injuries for pregnant women, including bruises, fractures, and head injuries. These injuries can have both immediate and long-term effects on a woman's health and well-being. For example, a pregnant woman who sustains a fracture may experience chronic pain or limited mobility, which can affect her ability to carry out daily activities or care for her baby after birth. Head injuries sustained during physical violence can also result in long-term neurological damage, which may affect a woman's cognitive abilities and mental health [12].

Additionally, physical injuries sustained during IPV can pose significant risks to the health and well-being of the developing fetus. For example, physical trauma to the abdomen can cause placental abruption, which can lead to hemorrhage, premature labor, and fetal distress. Pregnant women who experience physical violence may also be at higher risk of miscarriage or stillbirth [13]. In some cases, physical injuries sustained during IPV may require medical attention, such as hospitalization or surgery. Women who experience physical violence during pregnancy may also experience ongoing pain, disability, and reduced quality of life, which can have lasting effects on their overall health and well-being [14].

Maternal Mental Health

Maternal mental health is a critical aspect of overall maternal health and well-being. Intimate partner violence during pregnancy can lead to significant psychological trauma and mental health problems for pregnant women. Pregnant women who experience IPV are at increased risk of depression, anxiety, and PTSD. Depression is a common mental health problem experienced by pregnant women who have been exposed to IPV. It is characterized by feelings of sadness and hopelessness and a loss of interest in activities that were once enjoyable. Pregnant women with depression may also experience sleep disturbances, changes in appetite, and difficulty concentrating [15].

Anxiety is another common mental health problem experienced by pregnant women who have been exposed to IPV. It is characterized by excessive worry and fear and can include symptoms such as panic attacks, irritability, and difficulty sleeping. Anxiety can also lead to physical symptoms such as sweating, shaking, and shortness of breath. PTSD is a serious mental health condition that can occur after exposure to a traumatic event such as IPV. Pregnant women who have experienced IPV may develop symptoms of PTSD, such as flashbacks, nightmares, and feelings of intense fear and anxiety. These symptoms can interfere with daily life and have a significant impact on the health and well-being of the mother and her baby [16].

Maternal mental health problems can have long-lasting effects on the mother and can also impact the health and well-being of the baby. For example, depression during pregnancy has been linked to a higher risk of preterm birth and low birth weight. Infants born to mothers with untreated depression or anxiety may also be at increased risk of developmental delays and behavioral problems [17].

Reproductive Health Complications

Reproductive health complications are another adverse outcome associated with IPV during pregnancy. Research has shown that pregnant women who experience IPV are at increased risk for a range of reproductive health complications, including vaginal bleeding, premature rupture of membranes, and preterm labor. Vaginal bleeding can occur due to physical trauma or injuries sustained during violent episodes. This can lead to complications such as miscarriage, preterm labor, and low birth weight. Premature rupture of membranes, which occurs when the amniotic sac surrounding the baby breaks before the onset of labor, can also result from physical trauma. This can lead to premature delivery and an increased risk of infection for both the mother and the baby. Preterm labor can also be triggered by stress and trauma associated with IPV [18-20].

In addition to these complications, pregnant women who experience IPV may also be at increased risk for STIs, including HIV. This can occur as a result of forced or non-consensual sexual activity, a lack of access to protection, or a reluctance to seek medical care due to fear or shame [21].

Risk of Maternal Mortality

Intimate partner violence during pregnancy can have serious and potentially life-threatening consequences for pregnant women. The risk of maternal mortality, which refers to the death of a woman during pregnancy or within 42 days of the end of the pregnancy, can be significantly increased in cases of severe IPV [22]. Physical violence during pregnancy can result in serious injuries such as fractures, internal bleeding, and head trauma, which can lead to complications and, in some cases, death. Additionally, pregnant women who experience severe physical violence may be at risk of developing pregnancy-related complications such as preeclampsia, premature rupture of membranes, and preterm labor, which can also increase the risk of maternal mortality [23].

According to the World Health Organization (WHO), globally, an estimated 303,000 women died as a result of pregnancy- or childbirth-related complications in 2015. While the specific number of maternal deaths related to IPV during pregnancy is difficult to determine, research suggests that IPV is a significant risk factor for maternal mortality [6,9]. Given the serious risks associated with IPV during pregnancy, it is important for healthcare providers to routinely screen for IPV during prenatal care and provide appropriate interventions and resources for pregnant women who experience IPV. By addressing IPV during pregnancy, we can work to improve maternal and fetal health outcomes and reduce the risk of maternal mortality [24].

Adverse effects of IPV during pregnancy on fetal health

Intimate partner violence during pregnancy can have significant adverse effects on the health of the developing fetus. The following are some of the most commonly reported adverse effects of IPV during pregnancy on fetal health.

Low Birth Weight

Low birth weight is defined as a birth weight of less than 2,500 g (5.5 pounds) and is a common adverse outcome associated with IPV during pregnancy. Pregnant women who experience IPV are at an increased risk of delivering babies with LBW compared to women who do not experience IPV. The reasons for this association are not fully understood, but it is believed that IPV may directly or indirectly contribute to LBW through several mechanisms [10,25-27].

First, the stress and trauma of IPV during pregnancy can lead to increased levels of stress hormones, such as cortisol, in pregnant women. Prolonged exposure to stress hormones can negatively impact fetal growth and development, leading to LBW. Second, IPV during pregnancy may also be associated with poor maternal nutrition, inadequate prenatal care, and substance use, all of which can contribute to LBW. Additionally, IPV during pregnancy may increase the risk of premature birth, which is a known risk factor for LBW [10,25-27].

LBW is associated with an increased risk of infant mortality and long-term health problems for the baby. Infants with LBW are at increased risk of respiratory distress, infections, neurological disorders, developmental delays, and chronic diseases such as hypertension and diabetes in later life. These adverse health outcomes can have long-lasting effects on the physical, emotional, and cognitive development of the child and can impact their quality of life in adulthood [10,25-27].

Preterm Birth

Preterm birth is a serious health issue that can result in a range of health problems for the baby, including respiratory distress syndrome, jaundice, and infections. Preterm birth is a leading cause of neonatal mortality and morbidity worldwide [28,29]. Research has shown that IPV during pregnancy is associated with an increased risk of preterm birth. Women who experience IPV during pregnancy are more likely to deliver their babies prematurely compared to women who do not experience IPV. The risk of preterm birth is also higher among women who experience severe IPV, such as physical or sexual violence, compared to women who experience only psychological abuse [30].

The mechanisms underlying the association between IPV and preterm birth are not well understood. However, it is believed that the stress and trauma associated with IPV can trigger physiological responses that may increase the risk of preterm birth. For example, exposure to stress hormones like cortisol and adrenaline can lead to uterine contractions and preterm labor. Additionally, women who experience IPV may be more likely to engage in behaviors that increase the risk of preterm birth, such as smoking, drug use, or inadequate prenatal care [1,19,27].

The adverse effects of preterm birth can be serious and long-lasting. Infants born preterm are at increased risk of respiratory distress syndrome, jaundice, and infections, which can lead to hospitalization and long-term health problems. Preterm birth is also associated with an increased risk of developmental delays, cerebral palsy, and other neurological disorders [12,21].

Fetal Injury or Death

Fetal injury or death is one of the most serious consequences of IPV during pregnancy. When a pregnant woman experiences physical violence, the developing fetus is at risk of injury due to the force of the blows or trauma sustained by the mother [2-4,13]. Fetal injuries can include fractures, head injuries, or internal injuries that may affect the development and health of the fetus. In addition, some pregnant women who experience IPV may not receive adequate prenatal care, which can also contribute to adverse fetal outcomes such as low birth weight or premature birth [2,3,13].

In the most severe cases, IPV during pregnancy can result in fetal death. The trauma sustained by the mother can result in placental abruption. This can lead to fetal distress and death if not promptly addressed [12,23,30]. Fetal injury or death can have devastating consequences for the mother and her family, including psychological trauma, grief, and long-term health consequences. The impact of fetal injury or death on the mother's mental health and well-being should not be overlooked, and appropriate support and resources should be made available to help the mother cope with these difficult circumstances [9,19].

Long-Term Health Consequences for the Child

Children who are exposed to IPV during pregnancy may be at risk for a range of long-term health consequences, including behavioral and emotional problems, cognitive delays, and developmental delays [31,32].

Behavioral and emotional problems: Children who are exposed to IPV during pregnancy are at increased risk of developing behavioral and emotional problems, such as anxiety, depression, aggression, and antisocial behavior. These problems can persist into adolescence and adulthood, leading to difficulties in school, work, and social relationships [31,32].

Cognitive delays: Children who are exposed to IPV during pregnancy may be at risk for cognitive delays, such as problems with attention, memory, and language development. These delays can have long-term effects on academic achievement and overall cognitive functioning [31,32].

Developmental delays: Children who are exposed to IPV during pregnancy may also be at risk for developmental delays, such as delays in gross and fine motor skills, socialization, and self-care skills. These delays can affect a child's ability to function independently and may require ongoing support and intervention [3,4,18].

The long-term health consequences for the child can be attributed to several factors, including the impact of stress and trauma on the developing brain, disruptions to the parent-child relationship, and exposure to ongoing violence and instability in the home environment. Additionally, children who are exposed to IPV during pregnancy may be at increased risk of experiencing subsequent violence and trauma, further compounding the long-term health consequences [10,24,28].

Given the potential long-term health consequences for children exposed to IPV during pregnancy, it is critical to identify and address this issue as early as possible. Interventions and support services that promote healthy parent-child relationships, address trauma and stress, and promote cognitive and developmental skills can help mitigate the long-term effects of IPV on children [27].

Risk factors and protective factors for IPV during pregnancy

IPV during pregnancy is a complex issue that is influenced by a range of individual, relationship, community, and societal factors. Understanding these factors can help identify women who are at increased risk of experiencing IPV during pregnancy and develop effective interventions. The following are some of the most commonly reported risks and protective factors for IPV during pregnancy.

Individual Factors

Women who have a history of experiencing IPV or who are in an abusive relationship before becoming pregnant are at an increased risk of experiencing IPV during pregnancy. Additionally, women who have a low level of education or low income may be at a higher risk of experiencing IPV during pregnancy. Women who lack social support, such as family or friends, may also be more vulnerable to IPV during pregnancy [18,27]. Women who have low self-esteem may be more likely to believe that they deserve to be treated poorly or that they are not capable of leaving an abusive relationship. This can make it more difficult for them to seek help or take steps to protect themselves and their unborn child [21,32].

On the other hand, women who have strong coping skills and a high level of self-esteem may be more resilient and better able to cope with IPV. These women may be more likely to seek support from family or friends or to seek out resources such as counseling or legal assistance. Additionally, women who have a history of successfully coping with stressful situations may be better able to identify and respond to the warning signs of IPV during pregnancy [10,24].

Relationship Factors

The quality of the relationship between the pregnant woman and her partner can play a significant role in the risk of IPV during pregnancy. Women who have a partner with a history of IPV, substance abuse problems, or unemployment are at increased risk of experiencing IPV during pregnancy. In some cases, the pregnancy itself may trigger or exacerbate existing problems in the relationship, leading to an increase in IPV [1,31].

Research has also found that women who have a supportive partner, effective communication skills, and healthy conflict-resolution skills may be less likely to experience IPV during pregnancy. A partner who is supportive and engaged in the pregnancy may act as a protective factor, reducing the likelihood of IPV. Effective communication and conflict resolution skills can help couples manage conflicts and disagreements in a constructive and nonviolent way, reducing the risk of IPV [10,28].

Other relationship factors that may be relevant in the context of IPV during pregnancy include the length of the relationship, the age difference between partners, and the presence of children in the home. For example, some research has suggested that relationships with a large age difference between partners may be at increased risk of IPV during pregnancy. Additionally, with the presence of children in the home, their safety and well-being must also be considered [10,28].

Community and Societal Factors

Community and societal factors can have a significant impact on the risk of IPV during pregnancy. Women who live in communities with high rates of poverty, unemployment, and social inequality are at increased risk of experiencing IPV during pregnancy. Economic stress, lack of access to resources and services, and social isolation can all contribute to the occurrence of IPV [33].

Conversely, women who live in communities with strong social support networks, accessible healthcare, and comprehensive IPV prevention programs may be less likely to experience IPV during pregnancy. A community with social support networks, such as a local community center or neighborhood group, can provide women with a sense of connection and safety, as well as access to resources and services. Accessible healthcare can provide women with the opportunity to discuss any concerns about IPV with a healthcare provider and can also ensure that women receive appropriate medical care if they are injured as a result of IPV [5,17].

Comprehensive IPV prevention programs can also have a positive impact on reducing the occurrence of IPV during pregnancy. These programs can include public education campaigns, legal services, counseling services, and emergency shelters. Programs that focus on prevention, early intervention, and long-term support can be particularly effective in reducing the risk of IPV during pregnancy [12,32].

Screening and intervention for IPV during pregnancy

Screening for IPV during pregnancy is critical to identifying women who may be at risk and connecting them with appropriate interventions and resources. The following are some key considerations for screening and intervention for IPV during pregnancy.

The Importance of Screening for IPV During Prenatal Care

IPV during pregnancy is a significant public health issue that affects millions of women worldwide. Identifying IPV during pregnancy is critical to ensuring that pregnant women receive appropriate care and support. Routine screening for IPV during prenatal care is important because it allows healthcare providers to identify and address IPV early in pregnancy. This can help to prevent or mitigate the adverse effects of IPV on maternal and fetal health. Screening for IPV during prenatal care has been recommended by leading health organizations, including the American College of Obstetricians and Gynecologists (ACOG), because of the significant impact that IPV can have on maternal and fetal health [2,5,16,17].

During prenatal care visits, healthcare providers can use a variety of approaches to screen for IPV, including verbal screening using open-ended questions, standardized questionnaires, or computer-assisted self-interviews. These approaches have been shown to be effective in identifying IPV among pregnant women, and they can help to facilitate disclosure and increase access to appropriate resources and support [32].

In addition to identifying IPV, routine screening during prenatal care visits can also help to build trust and establish rapport between healthcare providers and pregnant women. It can help to create a safe and supportive environment in which pregnant women feel comfortable disclosing sensitive information and seeking help when needed. This can be especially important for women who are at increased risk of IPV due to factors such as poverty, social isolation, or a history of violence [12].

Approaches to Screening and Assessing for IPV

Verbal screening using open-ended questions: Healthcare providers can use verbal screening to ask pregnant women about their experiences of IPV in a supportive and non-judgmental manner. Open-ended questions, such as "Has anyone hurt you or made you feel afraid?" or "Is everything okay in your relationship?" can help to elicit information about possible IPV. Verbal screening is often the first step in identifying IPV and can be particularly helpful in cases where women may not feel comfortable disclosing IPV through other means [6,14,33].

Standardized questionnaires: Standardized questionnaires are validated tools that healthcare providers can use to screen for IPV during pregnancy. These questionnaires may ask about the frequency and severity of different types of IPV, such as physical violence, sexual violence, and emotional abuse. The most commonly used standardized questionnaires for screening IPV during pregnancy include the Abuse Assessment Screen and the Hurt, Insult, Threaten, and Scream (HITS) screening tool [6,14,33].

Computer-assisted self-interviews (CASI): CASI is a self-administered computer-based questionnaire that can be used to screen for IPV during pregnancy. CASI allows women to answer questions about their experiences of IPV in a private and confidential manner and has been found to be an effective screening tool. Some studies have suggested that women may be more likely to disclose IPV through CASI than through other means due to the increased sense of privacy and confidentiality it provides [6,7,14].

In addition to these screening approaches, healthcare providers should also be alert to signs of IPV during prenatal care, such as unexplained injuries or changes in behavior. Providers should ask follow-up questions if they suspect IPV and provide resources and support to women who disclose IPV. Healthcare providers should also be aware of the potential impact of cultural and linguistic barriers on screening and assessment for IPV and take steps to address these barriers in order to provide effective care to all pregnant women [19,31].

Interventions and Resources for Pregnant Women Experiencing IPV

Safety planning: Safety planning involves developing a personalized plan to help women identify and respond to potential safety risks related to IPV. This may include identifying safe places to go in an emergency, making a plan to leave an abusive situation, and creating a safety network of trusted friends, family, or advocates [6,8].

Counseling: Counseling can help women who have experienced IPV address the emotional and psychological impact of the violence. Counseling may be individual, group, or couple-based and may focus on a range of issues, such as coping with trauma, managing stress and anxiety, improving communication and conflict resolution skills, and enhancing self-esteem [6,8].

Advocacy: Advocacy involves providing women with support and resources to help them navigate the healthcare, legal, and social service systems. Advocates may help women with tasks such as obtaining restraining orders, finding safe housing, and accessing public benefits [6,8].

Legal assistance: Legal assistance can help women who have experienced IPV obtain legal protection, such as restraining orders, and navigate the legal system. Legal assistance may also include assistance with custody and divorce proceedings [1,31].

Referral to community resources such as shelters and hotlines: Community resources such as shelters and hotlines can provide women with a safe place to stay, crisis intervention services, and emotional support. Shelters may offer a range of services, such as counseling, legal advocacy, and case management [12,23].

Regular follow-up: Healthcare providers should follow up regularly with pregnant women who have experienced IPV to monitor their safety and well-being. Follow-up may involve checking in on women's physical and emotional health, ensuring that they are connected with appropriate resources and interventions, and monitoring for potential safety risks [12,23].

Overall, interventions and resources for pregnant women experiencing IPV should be comprehensive and tailored to meet individual needs. Healthcare providers, social service agencies, and community organizations should work collaboratively to ensure that women who have experienced IPV have access to a range of services and support that can help them address the physical, emotional, and psychological impacts of the violence.

## Conclusions

Intimate partner violence during pregnancy is a serious public health issue that can have significant adverse effects on the health and well-being of both mothers and their babies. Maternal and fetal health outcomes can be affected by a range of factors, including the type, frequency, and severity of the violence, as well as the availability of resources and interventions. This review has highlighted the adverse effects of IPV during pregnancy on maternal and fetal health, including physical injuries, maternal mental health, reproductive health complications, low birth weight, preterm birth, fetal injury or death, and long-term health consequences for the child. It has also outlined risk and protective factors for IPV during pregnancy and approaches to screening and intervention. To effectively address IPV during pregnancy, it is important to recognize the complex interplay of individual, relationship, community, and societal factors that contribute to this issue. Future research should focus on identifying effective interventions that can address IPV during pregnancy and improve maternal and fetal health outcomes. Additionally, healthcare providers, policymakers, and community leaders should work together to increase awareness and understanding of IPV during pregnancy and ensure that pregnant women who experience IPV have access to appropriate resources and support.
